# Deficiency of Parkinson’s Related Protein DJ-1 Alters Cdk5 Signalling and Induces Neuronal Death by Aberrant Cell Cycle Re-entry

**DOI:** 10.1007/s10571-022-01206-7

**Published:** 2022-02-19

**Authors:** María José López-Grueso, Carmen Alicia Padilla, José Antonio Bárcena, Raquel Requejo-Aguilar

**Affiliations:** 1 Department of Biochemistry and Molecular Biology, University of Córdoba, 14071 Córdoba, Spain; 2 Maimónides Biomedical Research Institute of Córdoba (IMIBIC), 14071 Córdoba, Spain

**Keywords:** DJ-1, Tau, Cell cycle, Neuronal death, Parkinson disease

## Abstract

**Abstract:**

DJ-1 is a multifunctional protein involved in Parkinson disease (PD) that can act as antioxidant, molecular chaperone, protease, glyoxalase, and transcriptional regulator. However, the exact mechanism by which DJ-1 dysfunction contributes to development of Parkinson’s disease remains elusive. Here, using a comparative proteomic analysis between wild-type cortical neurons and neurons lacking DJ-1 (data available via ProteomeXchange, identifier PXD029351), we show that this protein is involved in cell cycle checkpoints disruption. We detect increased amount of p-tau and α-synuclein proteins, altered phosphoinositide-3-kinase/protein kinase B (PI3K/AKT) and mitogen-activated protein kinase (MAPK) signalling pathways, and deregulation of cyclin-dependent kinase 5 (Cdk5). Cdk5 is normally involved in dendritic growth, axon formation, and the establishment of synapses, but can also contribute to cell cycle progression in pathological conditions. In addition, we observed a decrease in proteasomal activity, probably due to tau phosphorylation that can also lead to activation of mitogenic signalling pathways. Taken together, our findings indicate, for the first time, that aborted cell cycle re-entry could be at the onset of DJ-1-associated PD. Therefore, new approaches targeting cell cycle re-entry can be envisaged to improve current therapeutic strategies.

**Graphical Abstract:**

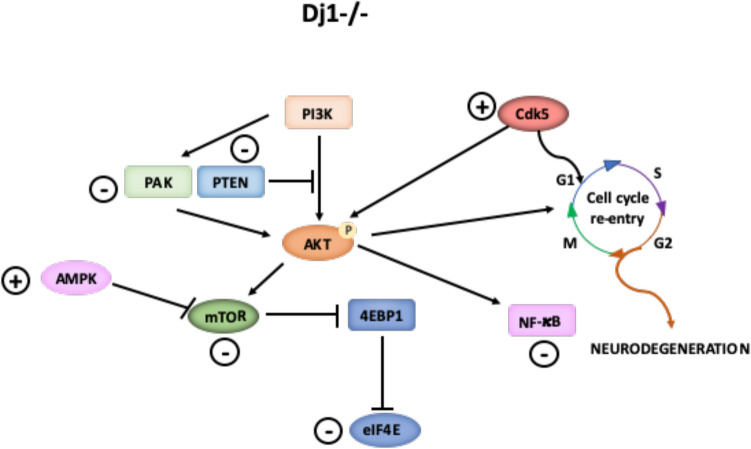

**Supplementary Information:**

The online version contains supplementary material available at 10.1007/s10571-022-01206-7.

## Introduction

DJ-1 protein was first described as the product of an oncogene and its high expression has been reported in many cancers (Nagakubo et al. 1997). However, DJ-1 has also been linked to a familiar form of Parkinson’s disease (PD) as well as other neurodegenerative diseases, such as Alzheimer disease, Huntington disease, and amyotrophic lateral sclerosis (Bonifati et al. 2003; Ariga 2017). The extensive number of disorders in which DJ-1 is involved, and the fact that its function remains still unclear, makes it difficult to know its exact mechanism of action. However, increased levels of oxidative stress associated with DJ-1 deficiency seem to play a central role in neurodegeneration (Ariga 2017). DJ-1 can act as an antioxidant both directly by scavenging reactive oxygen species and through regulation of proteins and transcription factors as the nuclear factor erythroid 2-related factor 2 (Nrf2). DJ-1 stabilizes Nrf2 by preventing association with its inhibitor protein, Keap1, once activated Nrf2 up-regulates antioxidant gene expression, which in turn decrease oxidative stress (Clements et al. 2006). Emerging evidence indicate that aberrant cell cycle associated to oxidative stress in mature neurons may be behind neurodegeneration. Expression of cell cycle markers in differentiated neurons is necessary to carry out synaptic function, neuronal migration, and maturation (Lim and Kaldis 2013). However, overexpression of these markers can also lead to a cell cycle re-entry, lethal to neurons, through different pathways. Activation of cell cycle can be triggered by ROS through oxidative stress-induced DNA damage, ubiquitin proteasome system (UPS) dysfunction, accumulation of toxic proteins aggregates composed of α-synuclein, parkin, tau, and amyloid β, and activation of signalling pathways, including phosphatidylinositol 3-kinase (PI3K)/Protein Kinase B (Akt), p38 mitogen-activated protein kinases (MAPKs), Wnt/β-catenin, and Notch (Sharma et al. 2017). Interestingly DJ-1 has been associated to many of these events. Thus, loss of function or mutation of DJ-1 can lead to α-synuclein accumulation and aggregation along with an increase in tau hyperphosphorylation (Xu et al. 2017; Wang et al. 2013; Chen et al. 2021). The increase of tau phosphorylation and consequent dysfunction of ubiquitin–proteasome system (UPS) activates mitogenic signalling pathways that induce cell cycle progression (Sharma et al. 2017). DJ-1 also participates in the regulation of signalling pathways inhibiting apoptosis through p38-MAPK pathway and activating PI3K/Akt pathway to mediate cell survival and proliferation (Oh and Mouradian 2017). DJ-1 lack could affect these pathways leading to cell cycle re-entry and apoptosis by tau hyperphosphorylation (Wang et al. 2013; Sharma et al. 2017). Finally, DJ-1 has been described to regulate proteasomal activity depending on its oxidation state (Moscovitz et al. 2015); absence of DJ-1 could provoke UPS dysfunction and cyclins accumulation, inducing post-mitotic cell division.

Thereby, it would not be surprising that lack of DJ-1 could be related to an abnormal activation of the cell cycle, although this has not been previously described. We have observed that fibroblasts lacking DJ-1 showed increased proliferation (Requejo-Aguilar et al. 2015), and neurons and neural precursors of mice deficient in Pink1, a protein transcriptionally regulated by DJ-1, showed an increase in the number of cells re-entering the cell cycle (Requejo-Aguilar et al. 2014).

Here we combined biochemical, cellular, and proteomic approaches to study the effect of DJ-1 absence in primary cortical neuron cultures. Our results revealed that lack of DJ-1 up-regulates proteins related to cell cycle control and proliferation, protein translation, proteasome-mediated protein degradation, and phagosome maturation. These changes suggest, in a novel way, that lack of DJ-1 leads to cell cycle activation in mature neurons that could be responsible for their death and therefore contribute to the development of Parkinson’s disease.

## Results

### Differential Proteomic Profiles Between Wild-Type and Mutant DJ-1 Neurons

**Fig. 1:**
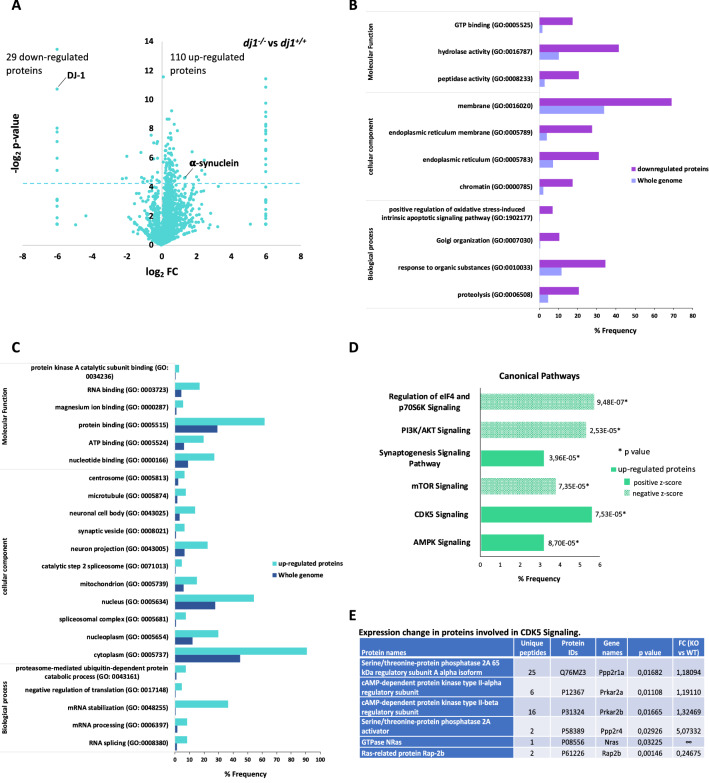
Label-free quantitative (LFQ) Proteomic analysis of changes in DJ-1 knockout (*dj1*^*−/−*^-KO) primary-cultured neurons. **A** Volcano plot showing the outcome of neurons *dj1*^*−/−*^ vs *dj1*^+*/*+^, *t* test performed with Perseus. Proteins are distributed along *x*-axis by fold change and *y*-axis by *p*-value; proteins over the dashed line represent those changing significantly in the absence of DJ-1. **B**–**E** Gene ontology and Ingenuity pathways enrichment analysis. Enrichment analysis was performed by GO terms using GOrilla, String and David tools, and Ingenuity Pathways Analysis tool (IPA). **B** and **C** show GO biological terms, cellular components, and molecular functions clusters that are down- or up-regulated in neurons lacking DJ-1 protein, respectively. **D** shows canonical pathways more significantly altered in DJ-1-deficient neurons detected by IPA indicating induction or inhibition for the different pathways identified. Table **E** includes proteins related to CDK5 signalling that shows expression change in *dj1*^*−/−*^ neurons compared with wild-type

A comparative proteomic study was carried out to determine the effect of DJ-1 absence on the proteome of cortical neurons. After data filtering and analysis, a total of 2355 proteins were identified (Table S1) of which 140 showed significant variation (Table S2), most of them (~ 80%) being up-regulated in neurons knockout for DJ-1 (Fig. 1A, C). The absence of the spot corresponding to DJ-1 in knockout neurons is a proof of analysis reliability (Fig. 1A). The enrichment analysis, based on GO terms (significant differences in the frequency of the proteins identified relative to their frequency in the whole genome), showed that the most remarkable decreases were detected in proteins involved in proteolysis; induction of apoptosis; signalling related to cell proliferation and survival ,such as p21-activated kinase (PAK), phosphatase and tensin homolog (PTEN) and nuclear factor-κB (NF-κB); mitochondrial dysfunction; and Parkinson’s disease (Fig. 1B, Supplementary Fig. 1A). In the same way, the main increases were detected in proteins involved in mRNA splicing, proteasome activity, regulation of translation, Akt and AMP-activated protein kinase (AMPK) signalling, and cell cycle checkpoints (Fig. 1C, Supplementary Fig. 1B). Within the proteins that act as cell cycle checkpoints the following were identified: Serine/threonine protein phosphatase 2A (PP2A) involved in G1/S transition, DNA synthesis, and mitotic initiation (Wlodarchak and Xing 2016); 14–3-3 *η* related to inactivation of cyclin B (Gardino and Yaffe 2011); and Histone deacetylase 2 (HDAC2) that specifically binds to the promoter of p21^WAF/CIP1^ inhibiting p21 expression and promoting cell cycle progression (Li et al. 2017). The pathways induced or inhibited by these proteins are shown in Fig. 1D.

Altogether, the changes observed are compatible with DJ-1 inducing abnormal entry into the cell cycle that would be responsible for neuronal death associated to PD onset.

### DJ-1 Deficiency in Primary Neuron Cultures is Associated to α-Synuclein and p-Tau Accumulation

**Fig. 2:**
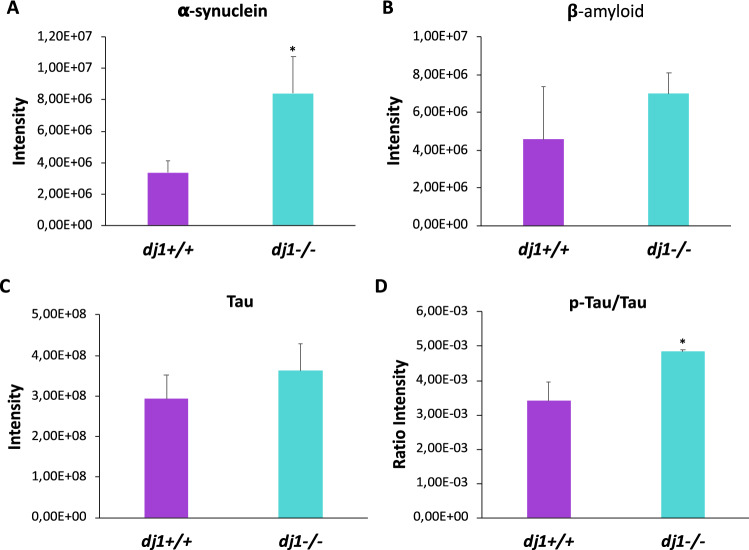
Quantitation of α-synuclein, β-amyloid, and tau in *dj1*^*−/−*^ neurons. Levels of α-synuclein (**A**), β-amyloid (**B**), and tau (**C**) were detected by mass spectrometry and statistically analysed using Perseus. Phosphorylated tau peptides were also analysed using MaxQuant and the ratio p-tau/tau was calculated (**D**). *Indicates significant differences with *p* < 0.05

Accumulation of unfolded protein-forming aggregates is a hallmark of most neurodegenerative diseases, including PD. Then, it is not surprising that DJ-1 deficiency has been associated with aggregation not only of α-synuclein but also of other proteins, such as p-tau (Xu et al. 2017; Wang et al. 2013; Chen et al. 2021). Our results show that DJ-1-deficient primary neurons have increased amount of both α-synuclein and p-tau and no significant increase in the amount of unphosphorylated tau and β-amyloid (Fig. 2A–D, Supplementary Fig. 2). Phosphorylation is present at tau residue T473 of *dj1*^*−/−*^ neurons which corresponds to T170 from mouse tau 430 isoform and to T181 from human tau 441 isoform.

These data indicate that DJ-1 might play a role in α-synuclein aggregation and tau phosphorylation.

### Tau Hyperphosphorylation in DJ-1 Knockout Neurons Seems to be Mediated by Cdk5 and Directly by Akt and AMPK

**Fig. 3:**
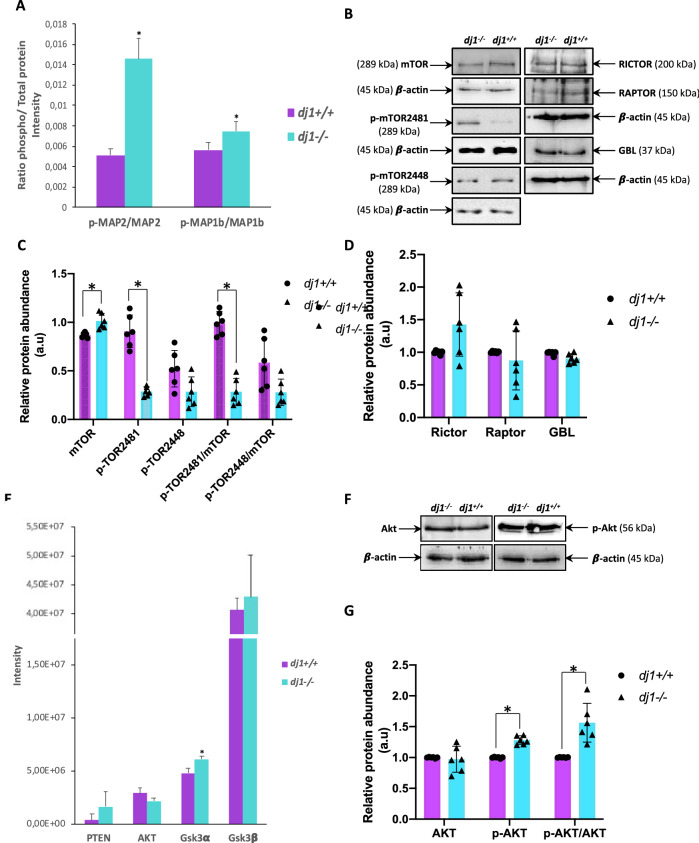
Analysis of proteins related to Cdk5, Akt/GSK, and mTOR pathways. **A** Ratio of phospho/total protein levels for the two targets of Cdk5 MAP2 and MAP1b as quantitated from the MS/MS spectra. **B**–**D** Western blot analysis and quantification of proteins belonging to mTOR pathway, including the two phosphorylated forms of mTOR, p-mTOR2481 (auto-phosphorylated residue), and p-mTOR2448 (phosphorylated by Akt), in neurons wild-type and knockouts for DJ-1 protein (a representative gel and global densitometry analysis are shown). **E** Levels of proteins related to Akt/GSK pathway identified by mass spectrometry. **F**, **G** Western blot analysis and quantification of Akt and phospho-Akt in neurons *dj1*^+*/*+^ and *dj1*^*−/−*^. Western blot data are mean ± SD (*N* ≥ 3; *t* test; *p < 0.05). Full-length blots are presented in Figs. S3 and S4

Cdk5 is a serine–threonine kinase that participates in cell cycle regulation and apoptotic neuronal death, and it is also involved in tau protein hyperphosphorylation (Kimura et al. 2014). Cdk5 also phosphorylates microtubule-associated protein 2 (MAP2) and microtubule-associated protein 1b (MAP1b) (Castro-Alvarez et al. 2014). Irregular Cdk5 activation and its phosphorylation of substrates as tau, MAP2, and MAP1b have been associated with pathological conditions, such as Alzheimer and Parkinson’s diseases. We have observed that proteins related to Cdk5 signalling were up-regulated in *dj1*^*−/−*^ neurons (Fig. 1D) in parallel with an increase in tau, MAP2, and MAP1b phosphorylation (Figs. 2D, 3A, Supplementary Fig. 2). p-tau could also be phosphorylated by another mechanism related directly to Akt, since canonical Akt and mTOR pathways are inhibited in *dj1*^*−/−*^ neurons (Figs. 1D, 3B–D). Some authors have described an increase of tau phosphorylation through Akt/Glycogen synthase kinase-3 beta (GSK-3β) pathway in neuroblastoma cells when DJ-1 was mutated (Wang et al. 2013). However, we did not observe changes in Akt/GSK-3β pathway since GSK-3β levels were similar in both *dj1*^+*/*+^ and *dj1*^*−/−*^ neurons (Fig. 3E) and no phosphorylation of this protein was detected. Nonetheless, Glycogen synthase kinase-3 alpha (GSK-3α) levels were significantly increased in the mutant (Fig. 3E) suggesting that this isoform might play a role in tau phosphorylation since it shares many substrates and functions with GSK-3β (Beurel et al. 2015). In fact, both GSK3α and β have been widely related with Alzheimer pathogenesis (Ma 2014) and both interact with tau (Sun et al. 2002). On the other hand, the increase in Akt phosphorylation level observed in DJ-1-knockout neurons (Fig. 3F, G) raises the possibility that Akt could phosphorylate tau, as has been described in vitro (Ksiezak-Reding et al. 2003). Finally, we found that AMPK signalling pathway was activated in *dj1*^*−/−*^ neurons (Supplementary Fig. 1B). AMPK is a serine–threonine kinase, which acts as energy sensor in eukaryotic cells and has been found to appear over-activated in patients affected by neurodegenerative disorders. AMPK has been involved in tau hyperphosphorylation. In vitro studies showed that AMPK could phosphorylate tau at several epitopes and in cellular models and was also found that AMPK phosphorylates tau under stress conditions (Domise and Vingtdeux 2016). AMPK activation in *dj1*^*−/−*^ neurons is consistent with the increase in p-tau observed in them.

### Factors that Trigger Cell Cycle Re-entry are Increased in DJ-1-Deficient Neurons

**Fig. 4:**
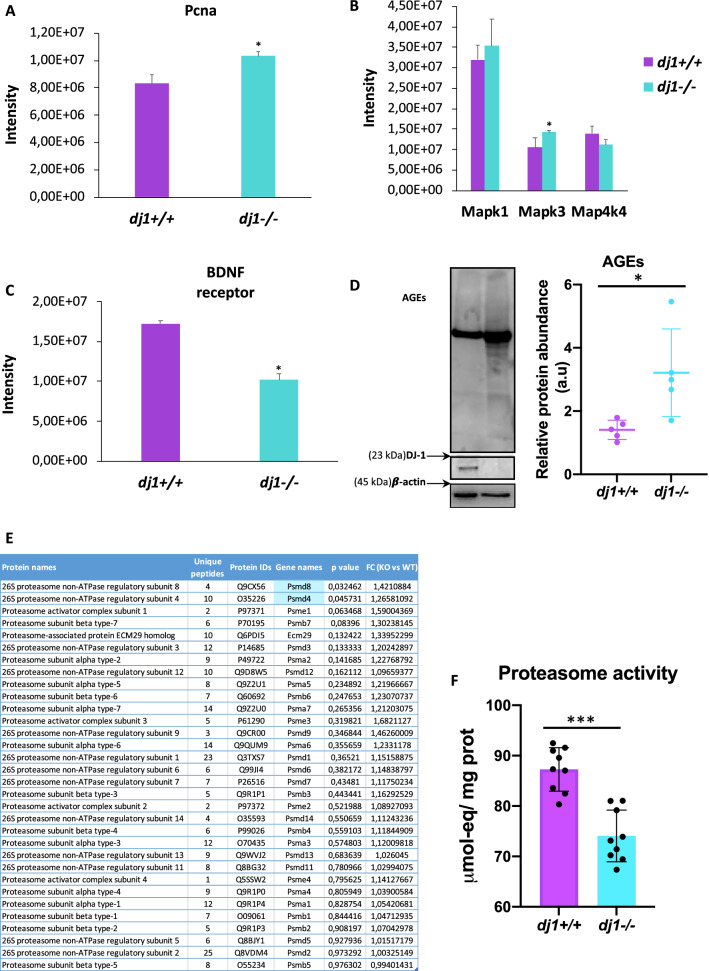
Analysis of cell cycle enhancing factors in neurons wild-type and *dj1*^*−/−*^. Levels of the protein PCNA implicated in cell cycle progression (**A**), MAPKs (**B**), and BDNF receptor (**C**) identified and quantified by mass spectrometry are shown. **D** Western blot and quantification of advanced glycation end products (AGEs) (a representative gel and global densitometry analysis are shown). **E** Proteasome proteins identified by mass spectrometry and the fold change *dj1*^*−/−*^ vs *dj1*^+*/*+^ are shown; highlighted are proteins with significant changes according to Perseus. **F** Proteasome activity was also determined in these neurons. Data are mean ± SD (*N* ≥ 3, *n* = 3; *t* test; *p < 0.05; ***p < 0.001). Full-length blots are presented in Fig. S5

Accumulation of α-synuclein and p-tau, and changes in Akt pathway, described above for *dj1*^*−/−*^ neurons, have also been described to force cell cycle re-entry in mature neurons leading to cell death as reported for PD and other neurodegenerative diseases (Sharma et al. 2017). In this regard, the role of Cdk5 should be highlighted because we observed an increase in Cdk5 signalling (Fig. 1D, E) and changes in cell cycle checkpoints in neurons deficient for DJ-1 (Supplementary Fig. 1B). Cdk5 is not only involved in tau hyperphosphorylation, but also in apoptotic neuronal death and cell cycle regulation. In mature neurons, Cdk5 is involved in axonal guidance and synaptic function, but in pathological conditions, such as accumulation of β-amyloid and prion peptides, it can induce proteins involved in reactivating cell cycle from G0 (Lopes et al. 2009; Veas-Perez de Tudela et al. 2015; Lapresa et al. 2019). Phospho-retinoblastoma and proliferating cell nuclear antigen (PCNA) are a cell cycle-related protein induced by Cdk5 (Lopes et al. 2009). Here, we show that *dj1*^*−/−*^ neurons have increased levels of PCNA (Fig. 4A) suggesting, as in previous reports, that neurons pass the S phase and die when they reach G2/M checkpoint. In addition, the proteomic analysis revealed an increase in other proteins in neurons lacking DJ-1 as shown for Mitogen-Activated Protein Kinase 3 (MAPK3) (Fig. 4B), which can support a transition from G1 to S phase. Moreover, a decrease in brain-derived neurotrophic factor (BDNF) receptor expression was observed in *dj1*^*−/−*^ neurons (Fig. 4C). BDNF has been described to downregulate cyclin B expression and Cyclin-dependent kinase 1 (Cdk1) activity in neurons, contributing to blockade of G2/M transition (Ovejero-Benito and Frade 2013). Thus, the decrease in BDNF receptor that we have observed in *dj1*^*−/−*^ neurons could release this blockade and stimulate cell cycle progression from G2 to M phase leading to cell death at this point of post-mitotic neurons.

Another factor contributing to cyclins expression and cell cycle re-entry is the production of advanced glycation end products (AGEs) (Kuhla et al. 2015). We analysed protein glycation and found that it was increased in neurons lacking DJ-1 (Fig. 4D). This is supported by the increase of glycolytic flux since is estimated that 0.1–0.4% of this flux results in methylglyoxal and therefore AGEs production (Allaman et al. 2015). Our results showed an increase in glucose consumption, lactate production, and higher activities of the glycolytic enzymes glyceraldehyde-3P dehydrogenase (GAPDH) and lactate dehydrogenase (LDH) resulting in the rise of methylglyoxal levels in *dj1*^*−/−*^ neurons (Supplementary Fig. 6). Therefore, AGEs increase in *dj1*^*−/−*^ neurons may be another factor contributing to cell cycle activation and neuronal death.

Finally, we detected up-regulation of some proteasome-related proteins (Fig. 1C), although the changes are minimal. We decided to investigate this pathway since loss of function of ubiquitin proteasome system (UPS) is involved in the pathogenesis of PD and other neurodegenerative diseases (Zheng et al. 2016). Additionally, tau phosphorylation leads to malfunction of UPS and cyclins accumulation that induce neurons re-entry into the cell cycle (Sharma et al. 2017). We observed a general up-regulation of proteasome proteins in neurons lacking DJ-1, although only two of them were significantly increased (Fig. 4E). This could be probably due to a compensatory mechanism since proteasome activity was decreased (Fig. 4F). Thus, loss of activity of UPS produced by the increase in tau phosphorylation, observed in *dj1*^*−/−*^ neurons, may contribute to cell cycle activation by cyclins that normally are degraded and consequently resulting in death of these neurons.

### DJ-1 Deficiency Induces Cell Cycle Re-entry and ROS Production Triggering Apoptosis in Post-mitotic Neurons

**Fig. 5:**
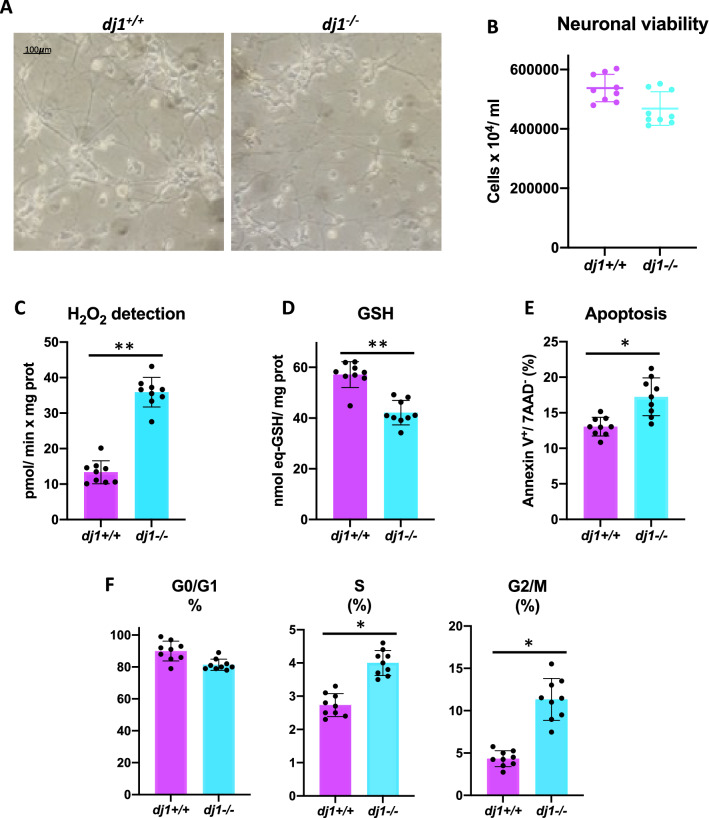
Effect of DJ-1 deficiency on cell cycle, ROS production, and apoptosis in post-mitotic neurons. **A** Phase-contrast microscopy of 8-day cultures mouse cortical neurons shows similar cell viability for wild-type and knockouts DJ-1 cells; this result was corroborated by cell counting (**B**). Lack of DJ-1 increases ROS production, as appreciated by rate of H_2_O_2_ detection (**C**), and decreases glutathione levels (**D**). The levels of apoptosis were also higher in neurons *dj1*^*−/−*^ as shown the proportion of annexin V-positive/7-AAD-negative cells, determined by flow cytometry (**E**). Incorporation of BrdU/7-AAD into DNA of cortical neurons shows an increase in the S and G2/M phases for cells knockouts (**F**). Data are expressed as mean ± SD (*N* ≥ 3, *n* = 3; *t* test; *p < 0.05; **p < 0.01)

We then assessed whether DJ-1 was involved in cell cycle re-entry and its effect in survival of cortical neurons. After 8 days of culture, neurons appear completely differentiated (Fig. 5A), and no differences in cell survival seem to be appreciated when cells are counted before performing the experiments (Fig. 5B), although a small decrease in the number of cells is seen in DJ-1-deprived cells. However, as has been previously described (Ariga 2017; Irrcher et al. 2010; Yang et al. 2017), lack of DJ-1 results in production of H_2_O_2_ and depletion of reduced glutathione (Fig. 5C, D). Since the increase in ROS and depletion of GSH lead to an increase of oxidative stress that activates apoptotic cell death, we set out to determine apoptosis. DJ-1 deficiency produced a significant increase in apoptosis rate as shown by flow cytometry analysis of annexin V-positive cells (Fig. 5E). Finally, to confirm the results obtained by proteomic analysis, we next analysed the proportion of cell cycle-entering neurons (BrdU^+^). Cortical neurons maintained their ability to incorporate BrdU showing a slight decrease in the G0/G1 phase and a significative increase in S and G2/M phases (Fig. 5F). This indicates an enlargement of the S and G2/M phases in *dj1*^*−/−*^ cortical neurons, as previously described for mouse embryonic fibroblasts deficient in DJ-1 (Requejo-Aguilar et al. 2015), that has been linked to neurodegeneration (Sharma et al. 2017; Frade and Ovejero-Benito 2015; Nandakumar et al. 2021).

## Discussion

Our results point to cell cycle re-entry as the cause of neuronal death associated to DJ-1 deficiency. Many of the pathways we have observed changing in neurons lacking DJ-1 have been implicated in cell cycle re-entry in neurodegeneration and neuromuscular disorders, including Parkinson’s, Alzheimer, or Huntington diseases (Sharma et al. 2017). Furthermore, the idea of cell cycle re-entry as the cause of neuronal death associated to DJ-1 deficiency is supported by the fact that, as in our case, lack of DJ-1 produces oxidative stress (Ariga 2017), which is one of the main causes reported for inducing cell cycle abnormalities in post-mitotic neurons (Sharma et al. 2017).

The proteomic analysis reveals that DJ-1 deficiency alters cell cycle checkpoints through Cdk5 signalling up-regulation (Fig. 5). Cdk5 acts in post-mitotic neurons controlling functions, such as axonal guidance and synapse regulation (Lopes et al. 2009). However, under pathological conditions, as is the case in our experimental model of increased oxidative stress associated to DJ-1 deficiency, Cdk5 induces cell cycle proteins and forces mature neurons to reactivate it (Lopes et al. 2009; Veas-Perez de Tudela et al. 2015; Lapresa et al. 2019). The increase in PCNA observed in *dj1*^*−/−*^ neurons indicates a progression in the cell cycle at least until G2 phase in these neurons, in agreement with the model proposed for other pathologies (Lopes et al. 2009). This may be mediated by the increase in tau phosphorylation (Castro-Alvarez et al. 2014) that contributes to DNA damage and activation of mitogenic signals as well as cyclins and Cdks imbalance by UPS dysfunction (Sharma et al. 2017).

In fact, DJ-1 mutations have been related to tau hyperphosphorylation (Wang et al. 2013) and it has been widely described that DJ-1 colocalises with tau inclusions in brains from PD patients indicating that DJ-1 can act as a chaperone modulating the aggregation and toxicity of tau as has been demonstrated for other proteins that form inclusions, such as α-synuclein, mutant huntingtin, and microtubule-associated protein 1B (MAP1B) (Zondler et al. 2014; Wang et al. 2011; Repici and Giorgini 2019).

Here, we show how DJ-1-deficient neurons show increased levels of tau phosphorylation and decreased proteasome activity that, along with up-regulation of Cdk5 signalling, agree with re-entry into the cell cycle as a possible cause of neuronal death. The increased phosphorylation of tau in neurons lacking DJ-1 in T170 of mouse 430 isoform is equivalent to T181 of human 441 isoform. This residue has been described to be phosphorylated in both normal and pathological conditions (Morris et al. 2015). Furthermore, cdk5 activation seems to be mediating tau hyperphosphorylation in different residues, including T181, increasing neurofibrillary pathologies development (Noble et al. 2003), and conversely Cdk5 inhibition reduces tau phosphorylation in T181 which contributes to reduce tau-associated pathologies (Seo et al. 2017).

Neurodegeneration associated to cell cycle re-entry is also related to disturbances in signalling pathways involving proteins, such as Notch, Wnt, Akt, and MAPK (Sharma et al. 2017). AMPK and p-Akt have been related to tau phosphorylation (Domise and Vingtdeux 2016; Ksiezak-Reding et al. 2003) and MAPK signalling pathway is activated and initiates cell cycle in post-mitotic hippocampal neurons after a lesion by increased expression of cyclin D (Hernandez-Ortega and Arias 2012). Therefore, the up-regulation of AMPK signalling, inhibition of PI3K/Akt/mTOR signalling with the increase in Akt phosphorylation, and increase in MAPK3 (ERK1) protein that we observed in neurons lacking DJ-1 can account for an aberrant activation of the cell cycle.

AGEs are increased in neurons lacking DJ-1. AGEs are mostly produced from methylglyoxal, an inevitable by-product of glycolysis (Allaman et al. 2015). Mouse embryonic fibroblasts (MEFs) deficient for protein DJ-1 have been described to increase their glycolytic rate (Requejo-Aguilar et al. 2015) and the same seems to happen in our case. Thus, is not surprising that *dj1*^*−/−*^ neurons showed an increase in glycated proteins. The observed increase in AGEs in neurons lacking DJ-1 could also contribute to cell cycle activation since it has been shown that AGEs produced as a consequence of oxidative stress act as mitogenic compounds inducing expression of cyclin D and DNA replication in a mouse model of Alzheimer disease deficient for apolipoprotein E (Kuhla et al. 2015).

Finally, other triggering factors contributing to cell cycle activation, such as UPS dysfunction and deprivation of the brain-derived neurotrophic factor (BDNF) and its receptor (Sharma et al. 2017), were also found in *dj1*^*−/−*^ neurons, supporting the idea of cell cycle re-entry as the cause of neuronal death. All these results are supported by the fact that we observed an increased number of neurons entering the cell cycle being in S and G2/M phases. Thus, targeting aberrant cell cycle progression using cyclins and Cdks inhibitors or molecules that induce cell cycle arrest could be a promising therapeutic strategy for PD treatment.

## Methods

### Ethical Statement Regarding the Use of Animals

Mice were bred at the Animal Experimentation Unit of the University of Córdoba, and all protocols were approved by the Bioethics Committee of the University of Córdoba in accordance with the Spanish legislation (RD53/2013). DJ-1-knockout mice were generously donated by Juan P. Bolaños (University of Salamanca, Spain) (Flicek et al. 2011), back-crossed for at least eleven generations with C57Bl/6J WT mice for the experiments, and bred under homozygosis.

### Cell Cultures

Cortical neurons in primary culture were prepared from foetal (E15.5) *dj1*^*−/−*^ (DJ-1 KO) and *dj1*^+*/*+^ (WT) offspring. Cells were seeded at 1.8 × 10^5^ cells cm^−2^ in cell culture plastic dishes previously coated with poly-d-lysine (15 µg ml^−1^) in neurobasal medium containing 2% of B-27 supplement (Gibco Brl-Life Technologies, Grand Island, NJ, USA) and 2 mM l-glutamine. Neurons were incubated at 37 °C in a humidified 5% CO_2_-containing atmosphere; at 2nd day, the medium was replaced, and neurons were used on the seventh or 8th day in vitro.

### Sample Preparation

Cortical neurons grown for 7–8 days were harvested and lysed in 100 mM Tris–HCl buffer, pH 7.5, using a homogenization pestle for 1.5 ml tubes. The extracts were centrifuged, and the supernatants were stored at − 80 °C until use for mass spectrometry or Western blot analysis. For the determination of proteasome activity, the neuron pellet was homogenized with sea sand in Tris–HCl buffer (0.5 mM EDTA, 1 mM PMSF, 100 mM Tris–HCl, pH 7.5). The samples were centrifuged, and the supernatant fraction was collected for analysis.

### Mass Spectrometry

#### Sample Preparation, Digestion, and nLC-MS2 Analysis

Samples from supernatants were prepared and analysed in the Proteomics Facility at Research Support Central Service (SCAI), University of Cordoba. Protein extracts were cleaned up in 10% 1D SDS-PAGE. The gel was stained with Coomassie Blue and protein bands were cut off, diced, and kept in water until digestion. Gel dices were firstly distained and protein resuspended and reduced by addition of 20 mM dithiothreitol. The mixture was cooled down to room temperature and alkylated by addition of 40 mM iodoacetamide. Proteolytic digestion was performed by addition of Trypsin (Promega, Madison, WI) and stopped by addition of trifluoroacetic acid, and the digested samples were finally Speedvac dried. Nano-LC was performed in a Dionex Ultimate 3000 nano UPLC (Thermo Scientific) as described previously (Padilla et al. 2019).

#### Data Analysis

MaxQuant (v1.5.7.0) (Cox and Mann 2008) and Perseus (v1.5.6.0) (Tyanova et al. 2016) software were used to analyse the different MSe runs in triplicate. Proteins were identified by searching raw data against the mouse UniprotKB/Swiss-Prot protein database (February 2018 version). Carbamidomethylation of cysteines as fixed modification, and oxidation of methionine and phosphorylation (ST) (Y) as variable modifications, were set for the study. Cleavage specificity was by trypsin, allowing for a maximum of one missed cleavage, a mass tolerance of 10 ppm for precursors and 0.01 Da for fragment ions. The false discovery rate (FDR) cut-off for protein identification was 1%. Enabling the “match between runs” option allowed for identification transfer between samples. Similar proteins were grouped, and only unique peptides were used for quantification. Identified from reverse database or contaminant hit proteins were removed prior to further analysis. Finally, the resultant list was analysed according to the instructions of the software developers (Tyanova et al. 2016). The criteria for considering a differentially expressed protein were that it was identified and quantified using at least two unique peptides and had a *p* ≤ 0.05 value. (Supplementary Tables S1 and S2). The same criteria were used to identify peptide phosphorylation, including a value of posterior error probability (PEP) ≤ 0.05 (Supplementary Table S3). Functional enrichment analysis was carried out using Ingenuity Pathways Analysis tool (IPA-Ingenuity Systems, www.ingenuity.com) and the results were compared with other tools, such as GOrilla, David, and String (Eden et al. 2009; Huang et al. 2007; Szklarczyk et al. 2015). The mass spectrometry proteomics data have been deposited to the ProteomeXchange Consortium via the PRIDE (Perez-Riverol et al. 2019) partner repository with the dataset identifier PXD029351.

#### Western Blot

Cells were lysed in 50 mM Tris–HCl buffer, pH 7.5 supplemented with 2% sodium dodecyl sulphate (SDS), 2 mM EDTA, 2 mM EGTA, phosphatase inhibitor (50 mM NaF), and protease inhibitors (100 mM phenylmethylsulphonyl fluoride, 50 µg ml^−1^ amastatin, and 50 µg ml^−1^ leupeptin), stored on ice for 30 min and boiled for 10 min. Extracts (25–100 μg) were subjected to SDS-PAGE and blotted onto nitrocellulose membrane (GE Healthcare Life Sciences). After electrotransfer, membranes were blocked for 1 h with 5% non-fat milk (BioRad) solubilized in TBS-T (150 mM NaCl, 50 mM Tris, pH 7.5, 0.05% Tween-20). Primary antibodies were anti-AGEs (rabbit, 1:100 dilution, ref #ab23722, AbCam), anti-mTOR Pathway Antibody Sampler Kit (rabbit, 1:1000 dilution, ref #9964, Cell Signalling), anti-Akt (mouse, 1:1000 dilution, ref #sc-5298, Santa Cruz Biotechnology), anti-p-Akt (rabbit, 1:1000 dilution, ref #4060, Cell Signalling), anti-DJ-1 (rabbit, 1:2000 dilution, ref #PA1-46262, Invitrogen), and anti-β-actin (mouse, 1:2000 dilution, ref #sc-47778, Santa Cruz Biotechnologies); secondary antibodies were anti-rabbit IgG-peroxidase conjugate (goat, 1:40,000 dilution, ref #A0545, Sigma-Aldrich) and anti-mouse IgG-peroxidase conjugate (goat, 1:80,000 dilution, ref #A2554, Sigma-Aldrich). Incubations were carried out overnight and for 2 h, respectively. Signal detection was performed with an enhanced chemiluminescence kit (ECL Plus Western blotting detection reagent from GE Healthcare). Western blots were done at least in triplicate and represent independent replicate experiments. The protein abundances were measured by densitometry of the bands on the membranes using ImageJ 1.48u4 software (National Institutes of Health, USA) and normalized against the corresponding β-actin band.

#### Proteasome Activity Assay

Cells from three independent experiments were lysed as described above. The proteasomal activity was measured using the 20S Proteasome Activity Assay kit according to the manufacturer’s instructions (APT280, Chemicon, Millipore). Proteasome activity is expressed as AMC (7-amino-4-methylcoumarin) relative fluorescent units (RFU) with the signal of the fraction inhibited with Lactacystin, an inhibitor of the 20S proteasome β5 subunit provided in the kit, as a reference.

#### ROS and Glutathione Analyses

For ROS assessment in cortical neurons, whole cell-derived H_2_O_2_ was measured, in intact cells, using the fluorescent AmplexRed probe (Invitrogen), 1.5 × 10^4^ cells were incubated in 100 μM AmplexRed in Krebs–Ringer Phosphate buffer (145 mM NaCl, 5.7 mM Na_2_PO_4_, 4.86 mM KCl, 0.54 mM CaCl_2_, 1.22 mM MgSO_4_, and 5.5 mM glucose, pH 7.4), and the luminescence recorded for 45 min at 1-min intervals using a Fluoroskan Ascent FL (Thermo Scientific, Rockford, IL, USA) fluorimeter (excitation: 538 nm, emission: 604 nm), and the slopes were used for calculations.

Glutathione levels were also determined, in cortical neurons, by adding 1% (wt/vol) of sulfosalicylic acid and an equal volume of NaOH 0.1 M to the cells. Cell lysates were centrifuged at 13,000×*g* for 5 min at 4 °C, and the supernatants were used for the determination of total glutathione (GSH + 2xGSSG), as described previously (Griffith 1980).

#### Flow Cytometric Detection of Neuronal Death

APC/C-conjugated annexin V and 7-aminoactinomycin D (7-AAD) (Becton Dickinson Biosciences, San Jose, CA, USA) were used to determine quantitatively the percentage of apoptotic neurons by flow cytometry. Neurons were softly detached from the plates using PBS containing EDTA 1 mM. Then, cells were stained with annexin V-APC and 7-AAD, following the manufacturer’s instructions and were analysed on a FACScalibur flow cytometer (15 mW argon ion laser tuned at 488 nm) using the CellQuest software (BDB). Cells stained with annexin V-APC and negative for 7-AAD were considered apoptotic.

#### Cell Viability and Cell Cycle Analysis

After 8-day culture, neurons were detached as previously described for apoptosis determination. BrdU incorporation into DNA and cell cycle phase percentage were determined by flow cytometry. This was achieved after 3 h of incubation with 10 mg ml^−1^ BrdU using the APC BrdU Flow Kit (BD Biosciences), following the manufacturer’s instructions. Number of cells alive after the incubation period was also confirmed by direct counting under light microscopy. Cells were visualized using a phase contrast-inverted microscope (CK2-TR; Olympus) and D Ach 10×, LWD CD Ach 20× brightfield objectives (Olympus).

#### Measurement of Enzymatic Activities, Glucose, Lactate, and Methylglyoxal

Glycolytic enzymatic activities and metabolites were assayed in fresh cell lysates obtained homogenizing cells with a pellet pestle in phosphate buffer 100 mM containing EDTA 0.5 mM, pH 7.4. Lactate dehydrogenase (LDH) and Glyceraldehyde-3-phosphate dehydrogenase (GAPDH) were measured according to the method described by Fielek and Mohrenweiser (1979). Glucose and lactate concentration in the culture medium were determined by standard enzymatic colorimetric assays at 505 nm (Labkit, Chemelex, S.A., Spain). The concentration of methylglyoxal (MG) was estimated by the method of Wild et al. (2012). MG content was calculated using a standard curve of known concentrations.

#### Protein Determination

Protein concentrations were determined in the lysates or in parallel cell culture incubations after solubilization with 0.1 M NaOH. Protein concentrations were determined using a Pierce BCA Protein Assay kit (Thermo Scientific, Milan, Italy) with bovine serum albumin as a standard.

#### Statistical Analysis

GraphPad Prism 9.0.1 (GraphPad Software, Inc., San Diego, CA) was used to assess significant differences. All measurements were carried out in triplicates, at least, in three independent experiments, and the results are expressed as the mean ± standard deviation (SD). Two groups were compared, and statistical analysis of the results was performed by unpaired *t* test with Welch’s correction. In all cases, *p* < 0.05 was considered significant.

## Supplementary Information

Below is the link to the electronic supplementary material.

Supplementary file1 (PDF 108 kb) **Supplementary Figure 1.** Gene ontology and enrichment analyses. Enrichment analysis was performed by GO terms using GOrilla, String, and David tools. **A** and **B** show pathways down- and up-regulated, respectively, identified in KEGG pathway database and altered in DJ-1-deficient neurons.

Supplementary file2 (PDF 199 kb)** Supplementary Figure 2.** Phosphorylation status analysis of tau, Map1b, and Map2 in cortical neurons *dj1*^+*/*+^ and *dj1*^*−/−*^. Peptides containing phosphorylation sites were quantified using the software MaxQuant and Perseus. The peptides, phosphorylation sites, and statistical significance are shown in table. Fragmentation MS/MS spectra containing phosphorylation sites are shown; the different ionic *y* and *b* series are sketched on the sequence of each peptide highlighting the corresponding tyrosine or serine modification.

Supplementary file3 (PDF 298 kb)** Supplementary Figure 3.** Original Western Blot images for Fig. 3B. **Supplementary Figure 4.** Original Western Blot images for Fig. 3F. **Supplementary Figure 5.** Original Western Blot images for Fig. 4D.

Supplementary file4 (PDF 146 kb) **Supplementary Figure 6**. Metabolic changes and methylglyoxal production in primary cortical neurons lacking DJ-1. Loss of DJ-1 enhances glycolytic flux as seen by the increased of glucose consumption (**A**), lactate production (**B**), and the high activity of the glycolytic enzymes glyceraldehyde-3P dehydrogenase (GAPDH) (**C**) and lactate dehydrogenase (LDH) (**D**). This leads to rise methylglyoxal levels in DJ-1-deficient neurons (**E**). Data are expressed as mean ± SD (*N* ≥ 3, *n* = 3; *t* test; *p < 0.05; **p < 0.01; ***p < 0.001). **F** Proposed mechanism for the increase of methylglyoxal and AGEs as glycolytic by-products when DJ-1 is absent.

Supplementary file5 (XLSX 268 kb)

Supplementary file6 (XLSX 27 kb)

Supplementary file7 (XLSX 137 kb)

## Data Availability

All data generated or analysed during this study are included in this published article and its supplementary information files.

## References

[CR1] Allaman I, Belanger M, Magistretti PJ (2015) Methylglyoxal, the dark side of glycolysis. Front Neurosci 9:23. 10.3389/fnins.2015.00023 25709564 10.3389/fnins.2015.00023 PMC4321437

[CR2] Ariga H (2017) DJ-1/Park7 protein. Springer, Berlin, Heidelberg, New York

[CR3] Beurel E, Grieco SF, Jope RS (2015) Glycogen synthase kinase-3 (GSK3): regulation, actions, and diseases. Pharmacol Ther 148:114–131. 10.1016/j.pharmthera.2014.11.016 25435019 10.1016/j.pharmthera.2014.11.016 PMC4340754

[CR4] Bonifati V, Rizzu P, van Baren MJ, Schaap O, Breedveld GJ, Krieger E, Dekker MC, Squitieri F, Ibanez P, Joosse M, van Dongen JW, Vanacore N, van Swieten JC, Brice A, Meco G, van Duijn CM, Oostra BA, Heutink P (2003) Mutations in the DJ-1 gene associated with autosomal recessive early-onset parkinsonism. Science 299(5604):256–259. 10.1126/science.1077209 12446870 10.1126/science.1077209

[CR5] Castro-Alvarez JF, Uribe-Arias SA, Mejia-Raigosa D, Cardona-Gomez GP (2014) Cyclin-dependent kinase 5, a node protein in diminished tauopathy: a systems biology approach. Front Aging Neurosci 6:232. 10.3389/fnagi.2014.00232 25225483 10.3389/fnagi.2014.00232 PMC4150361

[CR6] Chen WP, Zhang G, Cheng ZJ, Gu XH, Li M, Liu X (2021) Inhibitor kappa B kinase beta, modulated by DJ-1/p-VHL, reduces phosphorylated tau (p-tau) accumulation via autophagy in Alzheimer’s disease model. Neuroscience 452:1–12. 10.1016/j.neuroscience.2020.10.005 33069779 10.1016/j.neuroscience.2020.10.005

[CR7] Clements CM, McNally RS, Conti BJ, Mak TW, Ting JP (2006) DJ-1, a cancer- and Parkinson’s disease-associated protein, stabilizes the antioxidant transcriptional master regulator Nrf2. Proc Natl Acad Sci USA 103(41):15091–15096. 10.1073/pnas.0607260103 17015834 10.1073/pnas.0607260103 PMC1586179

[CR8] Cox J, Mann M (2008) MaxQuant enables high peptide identification rates, individualized p.p.b.-range mass accuracies and proteome-wide protein quantification. Nat Biotechnol 26(12):1367–1372. 10.1038/nbt.1511 10.1038/nbt.1511 19029910

[CR9] Domise M, Vingtdeux V (2016) AMPK in neurodegenerative diseases. Exp Suppl 107:153–177. 10.1007/978-3-319-43589-3_7 27812980 10.1007/978-3-319-43589-3_7

[CR10] Eden E, Navon R, Steinfeld I, Lipson D, Yakhini Z (2009) GOrilla: a tool for discovery and visualization of enriched GO terms in ranked gene lists. BMC Bioinform 10:48. 10.1186/1471-2105-10-48 10.1186/1471-2105-10-48 PMC2644678 19192299

[CR11] Fielek S, Mohrenweiser HW (1979) Erythrocyte enzyme deficiencies assessed with a miniature centrifugal analyzer. Clin Chem 25(3):384–388 162438

[CR12] Flicek P, Amode MR, Barrell D, Beal K, Brent S, Chen Y, Clapham P, Coates G, Fairley S, Fitzgerald S, Gordon L, Hendrix M, Hourlier T, Johnson N, Kahari A, Keefe D, Keenan S, Kinsella R, Kokocinski F, Kulesha E, Larsson P, Longden I, McLaren W, Overduin B, Pritchard B, Riat HS, Rios D, Ritchie GR, Ruffier M, Schuster M, Sobral D, Spudich G, Tang YA, Trevanion S, Vandrovcova J, Vilella AJ, White S, Wilder SP, Zadissa A, Zamora J, Aken BL, Birney E, Cunningham F, Dunham I, Durbin R, Fernandez-Suarez XM, Herrero J, Hubbard TJ, Parker A, Proctor G, Vogel J, Searle SM (2011) Ensembl 2011. Nucleic Acids Res 39(Database issue):D800–D806. 10.1093/nar/gkq1064 10.1093/nar/gkq1064 PMC3013672 21045057

[CR13] Frade JM, Ovejero-Benito MC (2015) Neuronal cell cycle: the neuron itself and its circumstances. Cell Cycle 14(5):712–720. 10.1080/15384101.2015.1004937 25590687 10.1080/15384101.2015.1004937 PMC4418291

[CR14] Gardino AK, Yaffe MB (2011) 14-3-3 proteins as signaling integration points for cell cycle control and apoptosis. Semin Cell Dev Biol 22(7):688–695. 10.1016/j.semcdb.2011.09.008 21945648 10.1016/j.semcdb.2011.09.008 PMC3507455

[CR15] Griffith OW (1980) Determination of glutathione and glutathione disulfide using glutathione reductase and 2-vinylpyridine. Anal Biochem 106(1):207–212. 10.1016/0003-2697(80)90139-6 7416462 10.1016/0003-2697(80)90139-6

[CR16] Hernandez-Ortega K, Arias C (2012) ERK activation and expression of neuronal cell cycle markers in the hippocampus after entorhinal cortex lesion. J Neurosci Res 90(11):2116–2126. 10.1002/jnr.23106 22811014 10.1002/jnr.23106

[CR17] Huang DW, Sherman BT, Tan Q, Kir J, Liu D, Bryant D, Guo Y, Stephens R, Baseler MW, Lane HC, Lempicki RA (2007) DAVID bioinformatics resources: expanded annotation database and novel algorithms to better extract biology from large gene lists. Nucleic Acids Res 35(Web Server issue):W169–W175. 10.1093/nar/gkm415 10.1093/nar/gkm415 PMC1933169 17576678

[CR18] Irrcher I, Aleyasin H, Seifert EL, Hewitt SJ, Chhabra S, Phillips M, Lutz AK, Rousseaux MW, Bevilacqua L, Jahani-Asl A, Callaghan S, MacLaurin JG, Winklhofer KF, Rizzu P, Rippstein P, Kim RH, Chen CX, Fon EA, Slack RS, Harper ME, McBride HM, Mak TW, Park DS (2010) Loss of the Parkinson’s disease-linked gene DJ-1 perturbs mitochondrial dynamics. Hum Mol Genet 19(19):3734–3746. 10.1093/hmg/ddq288 20639397 10.1093/hmg/ddq288

[CR19] Kimura T, Ishiguro K, Hisanaga S (2014) Physiological and pathological phosphorylation of tau by Cdk5. Front Mol Neurosci 7:65. 10.3389/fnmol.2014.00065 25076872 10.3389/fnmol.2014.00065 PMC4097945

[CR20] Ksiezak-Reding H, Pyo HK, Feinstein B, Pasinetti GM (2003) Akt/PKB kinase phosphorylates separately Thr212 and Ser214 of tau protein in vitro. Biochim Biophys Acta 1639(3):159–168. 10.1016/j.bbadis.2003.09.001 14636947 10.1016/j.bbadis.2003.09.001

[CR21] Kuhla A, Ludwig SC, Kuhla B, Munch G, Vollmar B (2015) Advanced glycation end products are mitogenic signals and trigger cell cycle reentry of neurons in Alzheimer’s disease brain. Neurobiol Aging 36(2):753–761. 10.1016/j.neurobiolaging.2014.09.025 25448604 10.1016/j.neurobiolaging.2014.09.025

[CR22] Lapresa R, Agulla J, Sanchez-Moran I, Zamarreno R, Prieto E, Bolanos JP, Almeida A (2019) Amyloid-ss promotes neurotoxicity by Cdk5-induced p53 stabilization. Neuropharmacology 146:19–27. 10.1016/j.neuropharm.2018.11.019 30452955 10.1016/j.neuropharm.2018.11.019

[CR23] Li S, Wang F, Qu Y, Chen X, Gao M, Yang J, Zhang D, Zhang N, Li W, Liu H (2017) HDAC2 regulates cell proliferation, cell cycle progression and cell apoptosis in esophageal squamous cell carcinoma EC9706 cells. Oncol Lett 13(1):403–409. 10.3892/ol.2016.5436 28123574 10.3892/ol.2016.5436 PMC5244900

[CR24] Lim S, Kaldis P (2013) Cdks, cyclins and CKIs: roles beyond cell cycle regulation. Development 140(15):3079–3093. 10.1242/dev.091744 23861057 10.1242/dev.091744

[CR25] Lopes JP, Oliveira CR, Agostinho P (2009) Cdk5 acts as a mediator of neuronal cell cycle re-entry triggered by amyloid-beta and prion peptides. Cell Cycle 8(1):97–104. 10.4161/cc.8.1.7506 19158499 10.4161/cc.8.1.7506

[CR26] Ma T (2014) GSK3 in Alzheimer’s disease: mind the isoforms. J Alzheimer’s Dis 39(4):707–710. 10.3233/JAD-131661 24254703 10.3233/JAD-131661 PMC8009280

[CR27] Morris M, Knudsen GM, Maeda S, Trinidad JC, Ioanoviciu A, Burlingame AL, Mucke L (2015) Tau post-translational modifications in wild-type and human amyloid precursor protein transgenic mice. Nat Neurosci 18(8):1183–1189. 10.1038/nn.4067 26192747 10.1038/nn.4067 PMC8049446

[CR28] Moscovitz O, Ben-Nissan G, Fainer I, Pollack D, Mizrachi L, Sharon M (2015) The Parkinson’s-associated protein DJ-1 regulates the 20S proteasome. Nat Commun 6:6609. 10.1038/ncomms7609 25833141 10.1038/ncomms7609

[CR29] Nagakubo D, Taira T, Kitaura H, Ikeda M, Tamai K, Iguchi-Ariga SM, Ariga H (1997) DJ-1, a novel oncogene which transforms mouse NIH3T3 cells in cooperation with ras. Biochem Biophys Res Commun 231(2):509–513. 10.1006/bbrc.1997.6132 9070310 10.1006/bbrc.1997.6132

[CR30] Nandakumar S, Rozich E, Buttitta L (2021) Cell cycle re-entry in the nervous system: from polyploidy to neurodegeneration. Front Cell Dev Biol 9:698661. 10.3389/fcell.2021.698661 34249947 10.3389/fcell.2021.698661 PMC8264763

[CR31] Noble W, Olm V, Takata K, Casey E, Mary O, Meyerson J, Gaynor K, LaFrancois J, Wang L, Kondo T, Davies P, Burns M, Veeranna NR, Dickson D, Matsuoka Y, Ahlijanian M, Lau LF, Duff K (2003) Cdk5 is a key factor in tau aggregation and tangle formation in vivo. Neuron 38(4):555–565. 10.1016/s0896-6273(03)00259-9 12765608 10.1016/s0896-6273(03)00259-9

[CR32] Oh SE, Mouradian MM (2017) Regulation of signal transduction by DJ-1. Adv Exp Med Biol 1037:97–131. 10.1007/978-981-10-6583-5_8 29147906 10.1007/978-981-10-6583-5_8 PMC5821264

[CR33] Ovejero-Benito MC, Frade JM (2013) Brain-derived neurotrophic factor-dependent cdk1 inhibition prevents G2/M progression in differentiating tetraploid neurons. PLoS ONE 8(5):e64890. 10.1371/journal.pone.0064890 23741412 10.1371/journal.pone.0064890 PMC3669015

[CR34] Padilla CA, Barcena JA, Lopez-Grueso MJ, Requejo-Aguilar R (2019) The regulation of TORC1 pathway by the yeast chaperones Hsp31 is mediated by SFP1 and affects proteasomal activity. Biochim Biophys Acta 1863(3):534–546. 10.1016/j.bbagen.2018.12.011 10.1016/j.bbagen.2018.12.011 30578832

[CR35] Perez-Riverol Y, Csordas A, Bai J, Bernal-Llinares M, Hewapathirana S, Kundu DJ, Inuganti A, Griss J, Mayer G, Eisenacher M, Perez E, Uszkoreit J, Pfeuffer J, Sachsenberg T, Yilmaz S, Tiwary S, Cox J, Audain E, Walzer M, Jarnuczak AF, Ternent T, Brazma A, Vizcaino JA (2019) The PRIDE database and related tools and resources in 2019: improving support for quantification data. Nucleic Acids Res 47(D1):D442–D450. 10.1093/nar/gky1106 30395289 10.1093/nar/gky1106 PMC6323896

[CR36] Repici M, Giorgini F (2019) DJ-1 in Parkinson’s disease: clinical insights and therapeutic perspectives. J Clin Med. 10.3390/jcm8091377 31484320 10.3390/jcm8091377 PMC6780414

[CR37] Requejo-Aguilar R, Lopez-Fabuel I, Fernandez E, Martins LM, Almeida A, Bolanos JP (2014) PINK1 deficiency sustains cell proliferation by reprogramming glucose metabolism through HIF1. Nat Commun 5:4514. 10.1038/ncomms5514 25058378 10.1038/ncomms5514

[CR38] Requejo-Aguilar R, Lopez-Fabuel I, Jimenez-Blasco D, Fernandez E, Almeida A, Bolanos JP (2015) DJ1 represses glycolysis and cell proliferation by transcriptionally up-regulating Pink1. Biochem J 467(2):303–310. 10.1042/BJ20141025 25670069 10.1042/BJ20141025 PMC4385472

[CR39] Seo J, Kritskiy O, Watson LA, Barker SJ, Dey D, Raja WK, Lin YT, Ko T, Cho S, Penney J, Silva MC, Sheridan SD, Lucente D, Gusella JF, Dickerson BC, Haggarty SJ, Tsai LH (2017) Inhibition of p25/Cdk5 attenuates tauopathy in mouse and iPSC models of frontotemporal dementia. J Neurosci 37(41):9917–9924. 10.1523/JNEUROSCI.0621-17.2017 28912154 10.1523/JNEUROSCI.0621-17.2017 PMC5637118

[CR40] Sharma R, Kumar D, Jha NK, Jha SK, Ambasta RK, Kumar P (2017) Re-expression of cell cycle markers in aged neurons and muscles: whether cells should divide or die? Biochim Biophys Acta 1863(1):324–336. 10.1016/j.bbadis.2016.09.010 10.1016/j.bbadis.2016.09.010 27639832

[CR41] Sun W, Qureshi HY, Cafferty PW, Sobue K, Agarwal-Mawal A, Neufield KD, Paudel HK (2002) Glycogen synthase kinase-3beta is complexed with tau protein in brain microtubules. J Biol Chem 277(14):11933–11940. 10.1074/jbc.M107182200 11812770 10.1074/jbc.M107182200

[CR42] Szklarczyk D, Franceschini A, Wyder S, Forslund K, Heller D, Huerta-Cepas J, Simonovic M, Roth A, Santos A, Tsafou KP, Kuhn M, Bork P, Jensen LJ, von Mering C (2015) STRING v10: protein-protein interaction networks, integrated over the tree of life. Nucleic Acids Res 43(Database issue):D447–D452. 10.1093/nar/gku1003 10.1093/nar/gku1003 PMC4383874 25352553

[CR43] Tyanova S, Temu T, Cox J (2016) The MaxQuant computational platform for mass spectrometry-based shotgun proteomics. Nat Protoc 11(12):2301–2319. 10.1038/nprot.2016.136 27809316 10.1038/nprot.2016.136

[CR44] Veas-Perez de Tudela M, Maestre C, Delgado-Esteban M, Bolanos JP, Almeida A (2015) Cdk5-mediated inhibition of APC/C-Cdh1 switches on the cyclin D1-Cdk4-pRb pathway causing aberrant S-phase entry of postmitotic neurons. Sci Rep 5:18180. 10.1038/srep18180 26658992 10.1038/srep18180 PMC4674757

[CR45] Wang Z, Zhang Y, Zhang S, Guo Q, Tan Y, Wang X, Xiong R, Ding J, Chen S (2011) DJ-1 can inhibit microtubule associated protein 1 B formed aggregates. Mol Neurodegener 6:38. 10.1186/1750-1326-6-38 21645326 10.1186/1750-1326-6-38 PMC3120713

[CR46] Wang Y, Liu W, He X, Zhou F (2013) Parkinson’s disease-associated DJ-1 mutations increase abnormal phosphorylation of tau protein through Akt/GSK-3beta pathways. J Mol Neurosci 51(3):911–918. 10.1007/s12031-013-0099-0 23979838 10.1007/s12031-013-0099-0

[CR47] Wild R, Ooi L, Srikanth V, Munch G (2012) A quick, convenient and economical method for the reliable determination of methylglyoxal in millimolar concentrations: the N-acetyl-l-cysteine assay. Anal Bioanal Chem 403(9):2577–2581. 10.1007/s00216-012-6086-4 22580513 10.1007/s00216-012-6086-4

[CR48] Wlodarchak N, Xing Y (2016) PP2A as a master regulator of the cell cycle. Crit Rev Biochem Mol Biol 51(3):162–184. 10.3109/10409238.2016.1143913 26906453 10.3109/10409238.2016.1143913 PMC4905575

[CR49] Xu CY, Kang WY, Chen YM, Jiang TF, Zhang J, Zhang LN, Ding JQ, Liu J, Chen SD (2017) DJ-1 inhibits alpha-synuclein aggregation by regulating chaperone-mediated autophagy. Front Aging Neurosci 9:308. 10.3389/fnagi.2017.00308 29021755 10.3389/fnagi.2017.00308 PMC5623690

[CR50] Yang J, Kim MJ, Yoon W, Kim EY, Kim H, Lee Y, Min B, Kang KS, Son JH, Park HT, Chung J, Koh H (2017) Isocitrate protects DJ-1 null dopaminergic cells from oxidative stress through NADP+-dependent isocitrate dehydrogenase (IDH). PLoS Genet 13(8):e1006975. 10.1371/journal.pgen.1006975 28827794 10.1371/journal.pgen.1006975 PMC5578699

[CR51] Zheng Q, Huang T, Zhang L, Zhou Y, Luo H, Xu H, Wang X (2016) Dysregulation of ubiquitin-proteasome system in neurodegenerative diseases. Front Aging Neurosci 8:303. 10.3389/fnagi.2016.00303 28018215 10.3389/fnagi.2016.00303 PMC5156861

[CR52] Zondler L, Miller-Fleming L, Repici M, Goncalves S, Tenreiro S, Rosado-Ramos R, Betzer C, Straatman KR, Jensen PH, Giorgini F, Outeiro TF (2014) DJ-1 interactions with alpha-synuclein attenuate aggregation and cellular toxicity in models of Parkinson’s disease. Cell Death Dis 5:e1350. 10.1038/cddis.2014.307 25058424 10.1038/cddis.2014.307 PMC4123098

