# Supplementary figures and images for: Deficiency of Parkinson’s Related Protein DJ-1 Alters Cdk5 Signalling and Induces Neuronal Death by Aberrant Cell Cycle Re-entry
Source: Cell Mol Neurobiol. 2022 Feb 19;43(2):757–69. doi: 10.1007/s10571-022-01206-7 (PMC9958167; doi:10.1007/s10571-022-01206-7)

A

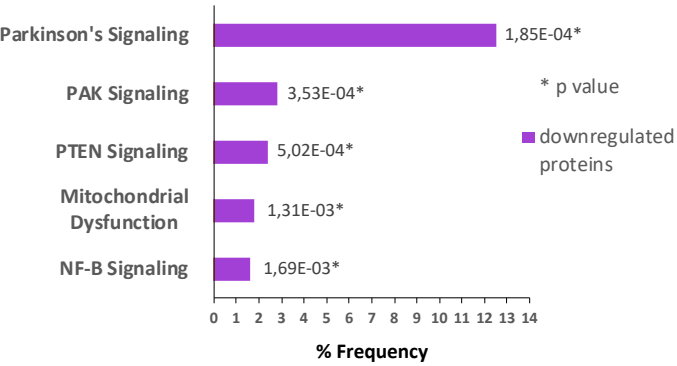

B

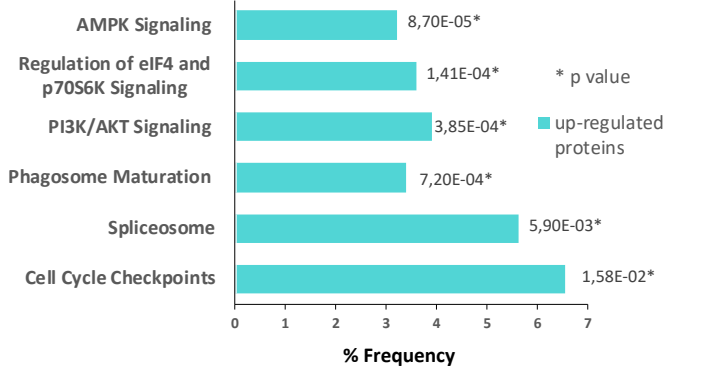

Supplement: Supplementary file 1 — Supplementary file1 (PDF 108 kb) Supplementary Figure 1. Gene ontology and enrichment analyses. Enrichment analysis was performed by GO terms using GOrilla, String, and David tools. A and B show pathways down- and up-regulated, respectively, identified in KEGG pathway database and altered in DJ-1-deficient neurons. [file 10571_2022_1206_MOESM1_ESM.pdf]

**A**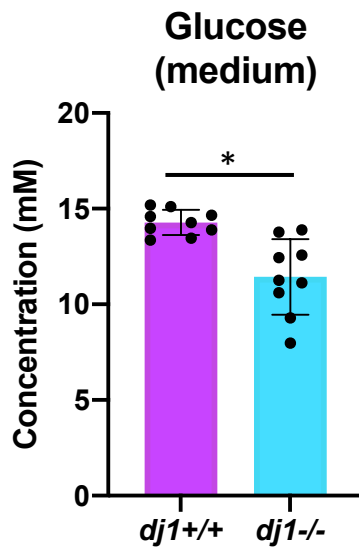**B**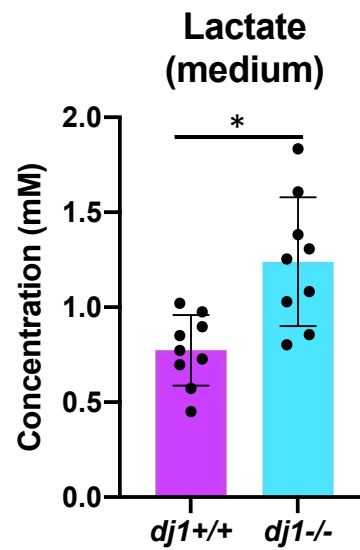**C**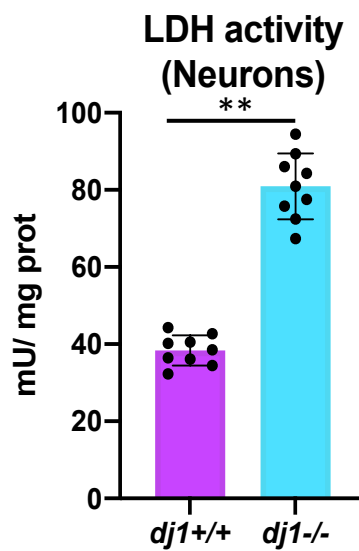**D**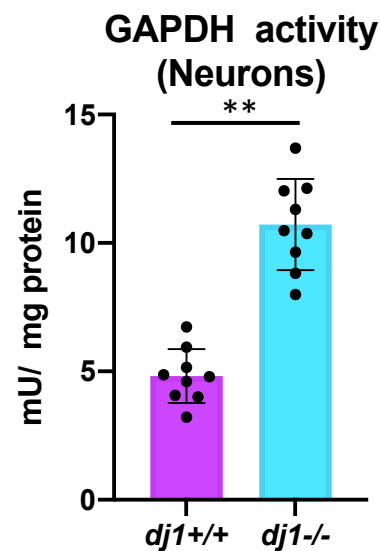**E**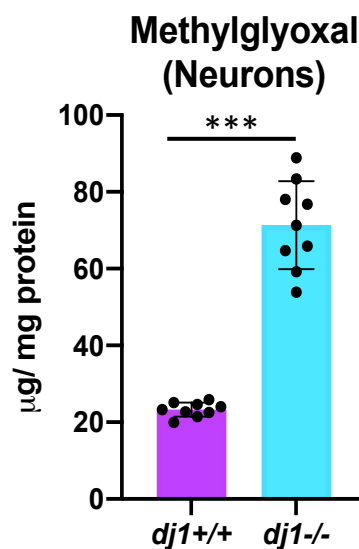**F**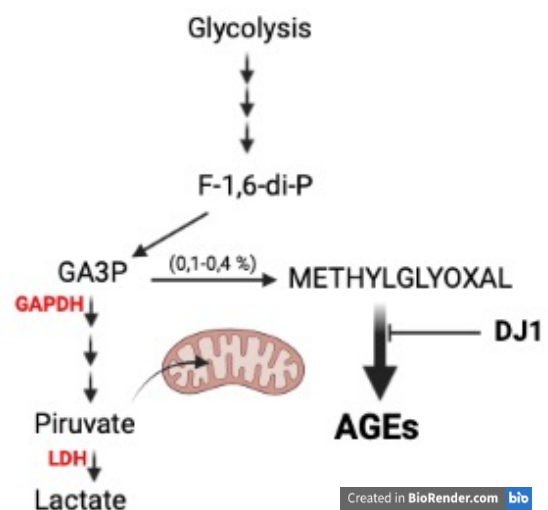

Supplement: Supplementary file 4 — Supplementary file4 (PDF 146 kb) Supplementary Figure 6. Metabolic changes and methylglyoxal production in primary cortical neurons lacking DJ-1. Loss of DJ-1 enhances glycolytic flux as seen by the increased of glucose consumption (A), lactate production (B), and the high activity of the glycolytic enzymes glyceraldehyde-3P dehydrogenase (GAPDH) (C) and lactate dehydrogenase (LDH) (D). This leads to rise methylglyoxal levels in DJ-1-deficient neurons (E). Data are expressed as mean ± SD (N ≥ 3, n = 3; t test; *p < 0.05; **p < 0.01; ***p < 0.001). F Proposed mechanism for the increase of methylglyoxal and AGEs as glycolytic by-products when DJ-1 is absent. [file 10571_2022_1206_MOESM4_ESM.pdf]
