# Supplementary material for: Deficiency of Parkinson’s Related Protein DJ-1 Alters Cdk5 Signalling and Induces Neuronal Death by Aberrant Cell Cycle Re-entry
Source: Cell Mol Neurobiol. 2022 Feb 19;43(2):757–69. doi: 10.1007/s10571-022-01206-7 (PMC9958167; doi:10.1007/s10571-022-01206-7)
Supplement: Supplementary file 3 — Supplementary file3 (PDF 298 kb) Supplementary Figure 3. Original Western Blot images for Fig. 3B. Supplementary Figure 4. Original Western Blot images for Fig. 3F. Supplementary Figure 5. Original Western Blot images for Fig. 4D. [file 10571_2022_1206_MOESM3_ESM.pdf]

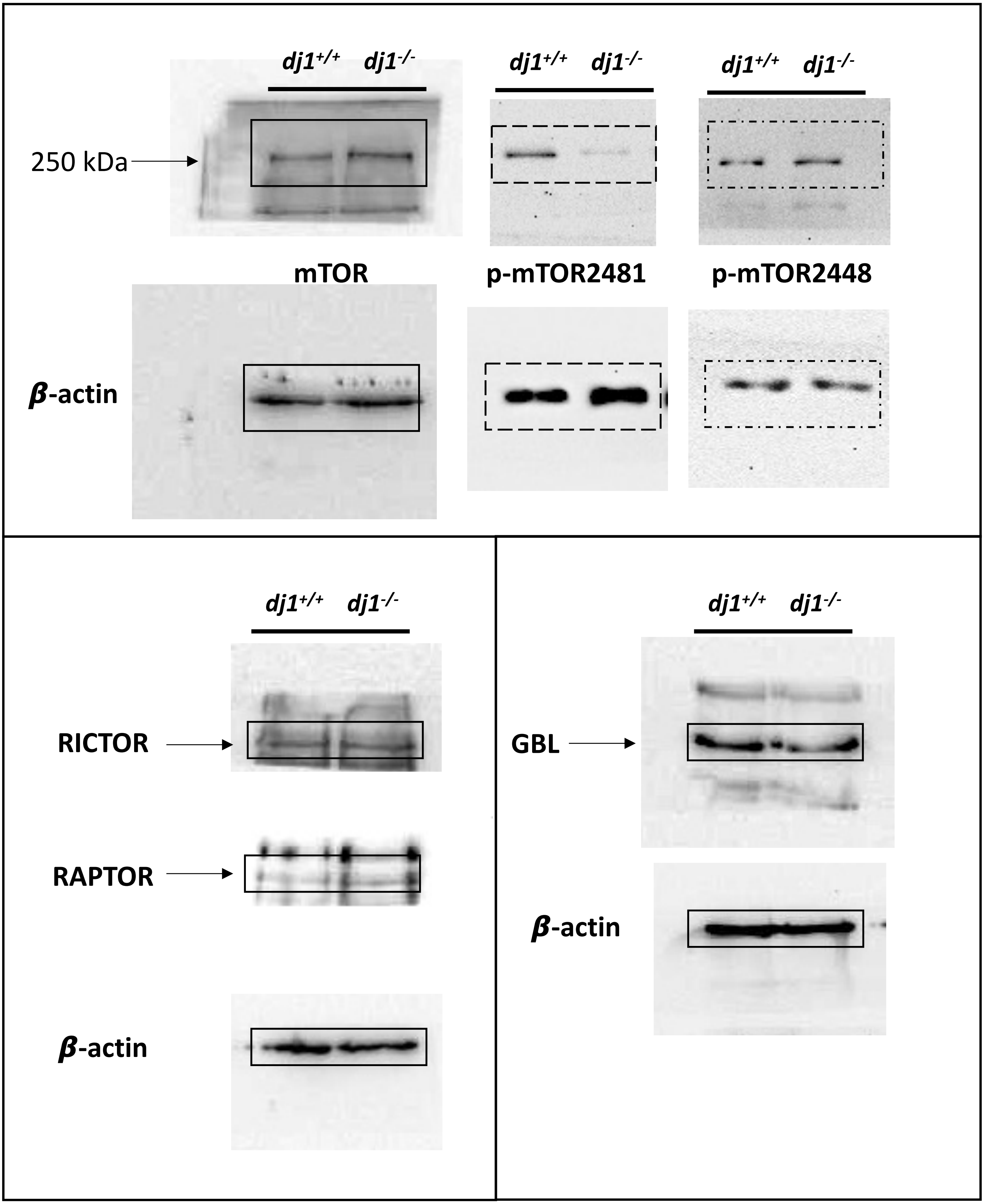

Figure S3. Original Western Blot images for Figure 3 B

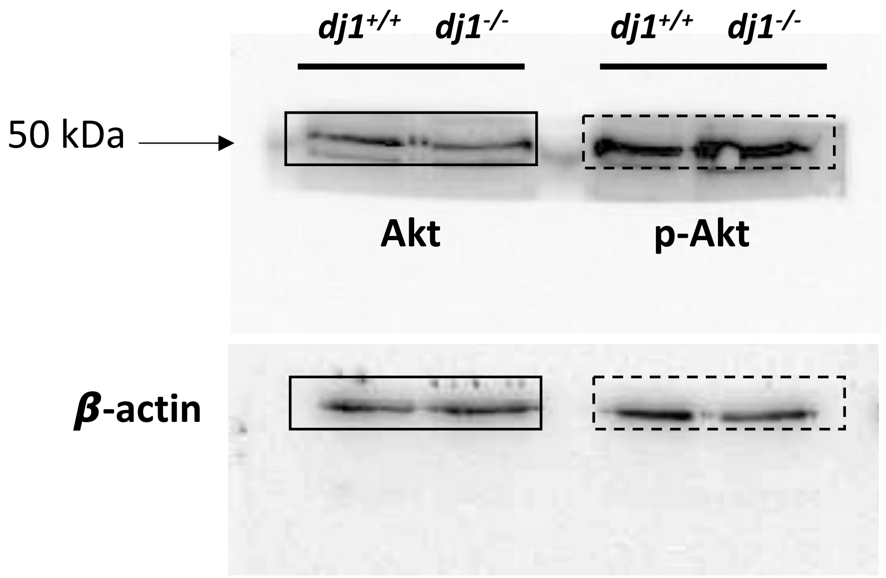

Figure S4. Original Western Blot images for Figure 3 F

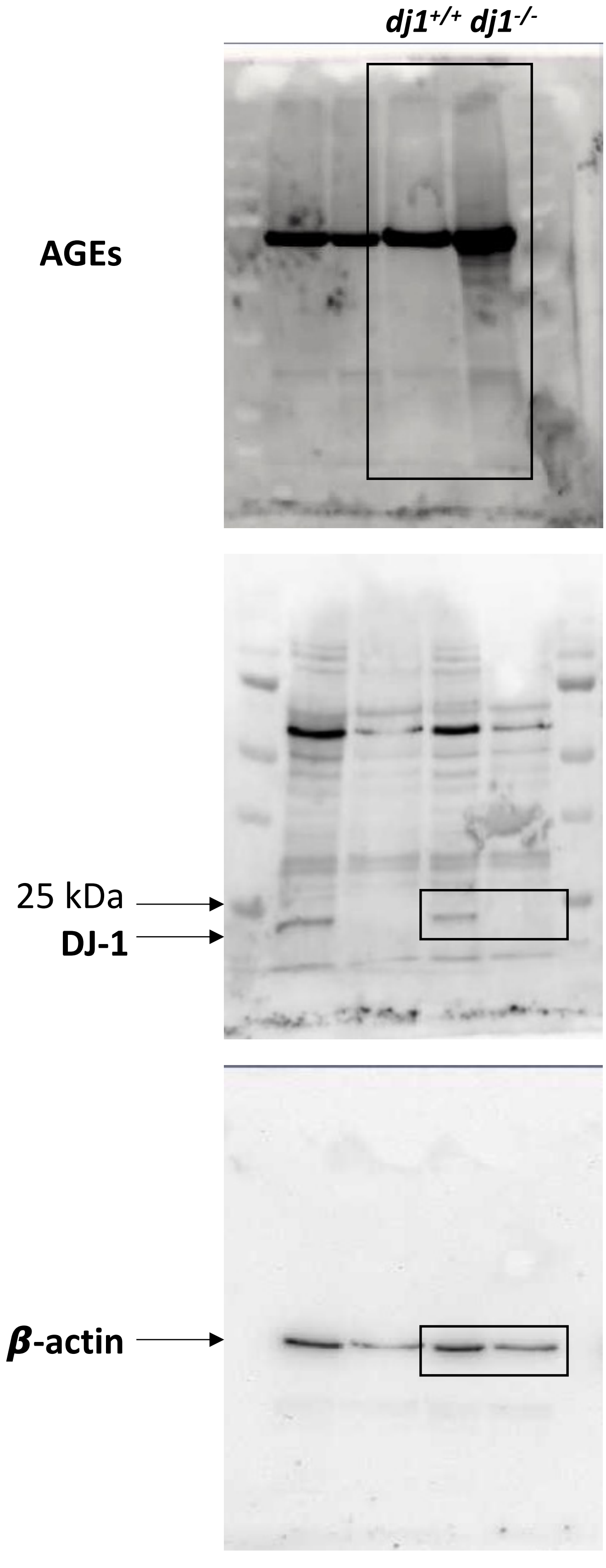

Figure S5. Original Western Blot images for Figure 4 D
