# Supplementary material for: Deficiency of Parkinson’s Related Protein DJ-1 Alters Cdk5 Signalling and Induces Neuronal Death by Aberrant Cell Cycle Re-entry
Source: Cell Mol Neurobiol. 2022 Feb 19;43(2):757–69. doi: 10.1007/s10571-022-01206-7 (PMC9958167; doi:10.1007/s10571-022-01206-7)

Gene name	Protein Name	Sequence	Modified Residue	Posterior error probability (PEP)
Mapt	Microtubule-associated protein tau	TTPSPKTPPGSGEPPKSGER	T(473)	3,04 x 10^{-4}
Map2	Microtubule-associated protein 2	VDHGAEIITQSPSR	S(1783)	9,31 x 10^{-21}
Map1b	Microtubule-associated protein 1B	TIKTPEDGGYTCEITEK	T(1928)	9,85 x 10^{-4}

TAU

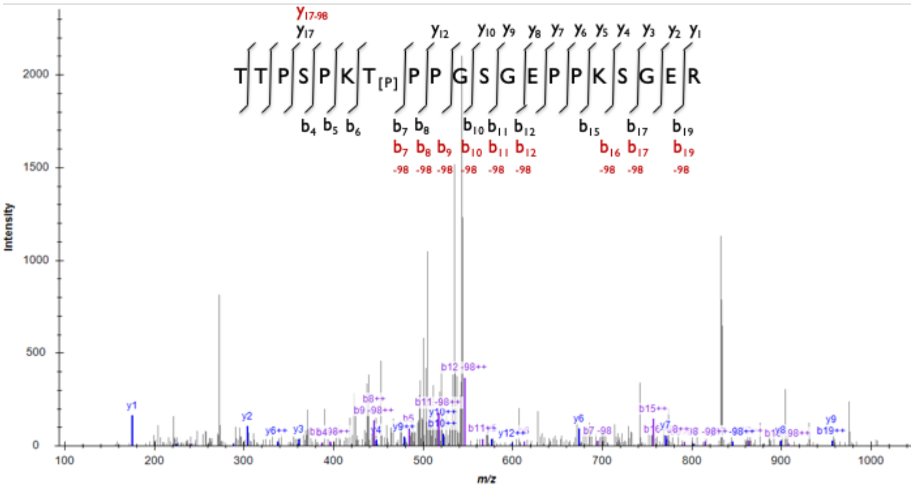

MAP2

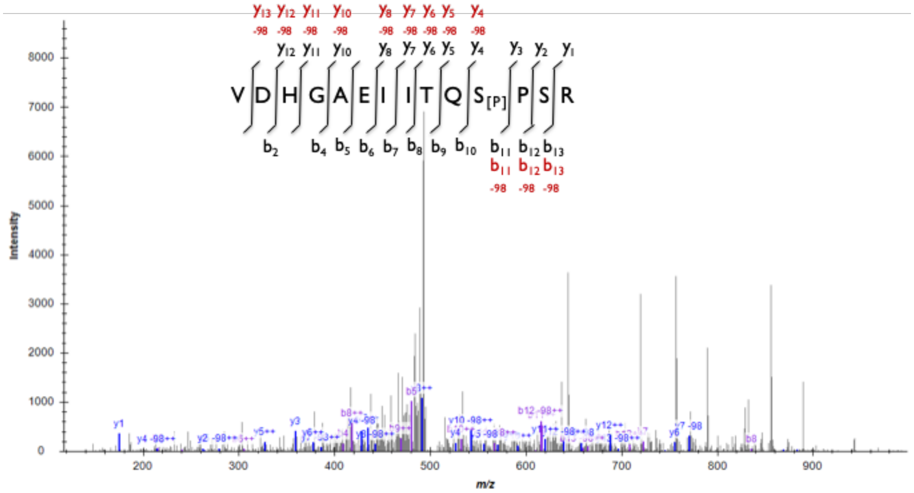

MAP1b

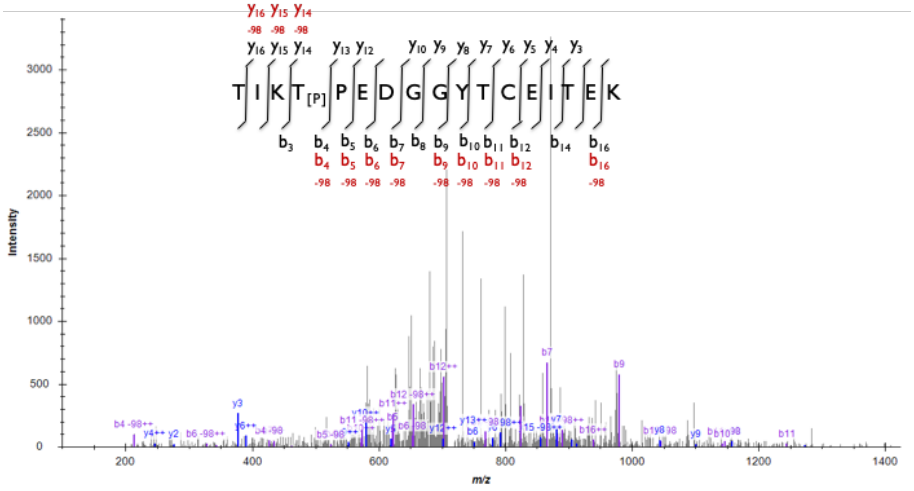

Supplement: Supplementary file 2 — Supplementary file2 (PDF 199 kb) Supplementary Figure 2. Phosphorylation status analysis of tau, Map1b, and Map2 in cortical neurons dj1+/+ and dj1−/−. Peptides containing phosphorylation sites were quantified using the software MaxQuant and Perseus. The peptides, phosphorylation sites, and statistical significance are shown in table. Fragmentation MS/MS spectra containing phosphorylation sites are shown; the different ionic y and b series are sketched on the sequence of each peptide highlighting the corresponding tyrosine or serine modification. [file 10571_2022_1206_MOESM2_ESM.pdf]
